# Ubiquitin signaling in cell cycle control and tumorigenesis

**DOI:** 10.1038/s41418-020-00648-0

**Published:** 2020-10-31

**Authors:** Fabin Dang, Li Nie, Wenyi Wei

**Affiliations:** 1 Department of Pathology, Beth Israel Deaconess Medical Center, Harvard Medical School, Boston, MA 02215 USA; 2 State Key Laboratory for Quality and Safety of Agro-products, School of Marine Sciences, Ningbo University, Ningbo, 315211 China

**Keywords:** Cancer, Ubiquitin ligases, Ubiquitylation

## Abstract

Cell cycle progression is a tightly regulated process by which DNA replicates and cell reproduces. The major driving force underlying cell cycle progression is the sequential activation of cyclin-dependent kinases (CDKs), which is achieved in part by the ubiquitin-mediated proteolysis of their cyclin partners and kinase inhibitors (CKIs). In eukaryotic cells, two families of E3 ubiquitin ligases, anaphase-promoting complex/cyclosome and Skp1-Cul1-F-box protein complex, are responsible for ubiquitination and proteasomal degradation of many of these CDK regulators, ensuring cell cycle progresses in a timely and precisely regulated manner. In the past couple of decades, accumulating evidence have demonstrated that the dysregulated cell cycle transition caused by inefficient proteolytic control leads to uncontrolled cell proliferation and finally results in tumorigenesis. Based upon this notion, targeting the E3 ubiquitin ligases involved in cell cycle regulation is expected to provide novel therapeutic strategies for cancer treatment. Thus, a better understanding of the diversity and complexity of ubiquitin signaling in cell cycle regulation will shed new light on the precise control of the cell cycle progression and guide anticancer drug development.

## Graphical abstract

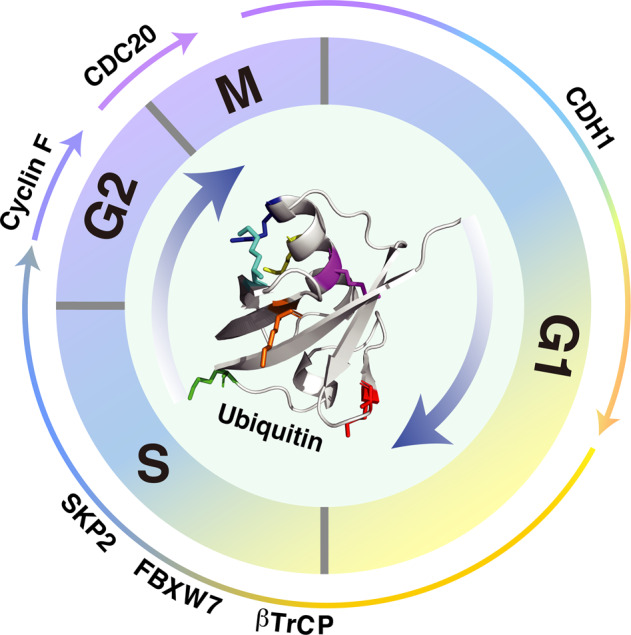

## Facts

1. The cell cycle is a tightly orchestrated cellular process that governs the timely DNA replication and cell division events.
2. Sequential activation of cyclin-dependent kinases (CDKs) drives cell cycle progression in a timely and precisely regulated manner.
3. The activity of CDKs is modulated by cyclin partners and CDK inhibitors (CKIs), which are tightly controlled by the ubiquitin–proteasome system.
4. Two important types of E3 ligases, the anaphase-promoting complex or cyclosome (APC/C) and Skp1-Cul1-F-box (SCF) complexes, are dedicated to cell cycle control.
5. Targeting E3 ubiquitin ligases provides effective therapeutic strategies for cancer treatment.

## Open questions

1. Unlike proteolytic signals, relatively little is known regarding the roles and mechanisms of non-proteolytic signals, such as the ones mediated by K6, K27, and K29 polyubiquitin chain, underlying the cell cycle control.
2. The specificity and diversity of deubiquitinating enzymes (DUBs) in regulating mitosis need to be further investigated.
3. How is the balance of ubiquitination–deubiquitination achieved to ensure accurate cell cycle progression remains elusive.
4. Unlike CDC20, the regulation of the enzymatic activity of CDH1 is not well defined yet.
5. The detailed mechanisms underlying spindle checkpoint imposed various E3 ligase activities of CDC20 toward different substrates need to be further investigated.
6. How those ubiquitination signaling events at the spindle checkpoint are integrated and orchestrated in a space–time-dependent manner remains not fully understood.

## Introduction

**Fig. 1:**
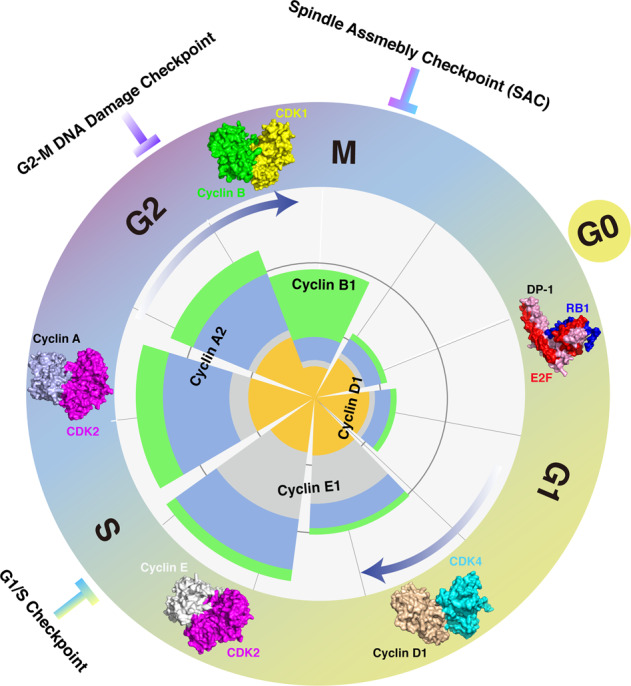
Overview of the mammalian cell cycle. The stages of the cell cycle are divided into four major phases: (1) G1 phase, also called the first gap phase. During the G1 phase, cells grow physically larger and duplicate cellular contents to prepare for the later steps; (2) S phase, cells synthesize a complete copy of DNA and duplicate the centrosome; (3) G2 phase, the second gap phase, cells grow more and prepare for mitosis; (4) M (mitotic) phase, during this phase, cells divide their copied DNA and cellular components, making two identical daughter cells. G0 phase is a quiescent stage that occurs outside of the cell cycle. During the G0 phase, cells are neither dividing nor preparing to divide. Sequential activation of Cyclin/CDKs drives cell cycle progression in a timely orchestrated manner. Briefly, Cyclin D1/CDK4 mainly functions in G1 phase to facilitate RB1 phosphorylation, releasing its suppression on E2F transcription factors; Cyclin E/CDK2 functions in S phase to control DNA replication; Cyclin A/CDK2 functions in later S phase to prepare the cell cycle entry into M phase; Cyclin B/CDK1 functions in M phase to be involved in regulation of chromatin separation. Additionally, three cell cycle checkpoints, G1/S checkpoint, G2-M DNA damage checkpoint, and spindle assembly checkpoint (SAC), are orchestrated to ensure the proper progression of the cell cycle. Protein structures of Cyclin/CDKs and RB1/E2F used here are as follows: RB1/E2F/DP (2AZE); Cyclin D1/CDK4 (2W9Z); Cyclin E/CDK2 (1W98); Cyclin A/CDK2 (6P3W); Cyclin B/CDK1 (4YC3).

The cell cycle is a series of tightly orchestrated molecular events that coordinately regulate DNA replication and chromosome segregation, eventually resulting in cell division and genetic material transmission. In eukaryotic cells, the cell cycle consists of four distinct phases, G1 phase (gap 1), S phase (DNA synthesis), G2 phase (gap 2), and M phase (mitotic) that proceed in a unidirectional manner (Fig. 1). The progression through each phase of the cell cycle is precisely regulated by a series of cyclin-dependent kinases (CDKs). The protein abundance of CDKs is constant, while their activities fluctuate throughout the cell cycle, which is mainly achieved by the periodic expression of cyclin coactivators and CDK inhibitors (CKIs). Briefly, in mid-to-late G1 phase, activation of Cyclin D-CDK4/6 complex mediates partial phosphorylation of the RB1 protein, releasing E2F transcription factors and thus allowing the expression of a set of genes that mediate cell cycle progression [1]. At the end of the G1 phase, the accumulation of Cyclin E activates CDK2 and promotes full phosphorylation of RB1 [2, 3], initiating cell cycle transition from G1 phase to S phase. As cell cycle enters S phase, Cyclin A, in replace of Cyclin E, associates with CDK2 to regulate the initiation of DNA replication and prevents the re-replication by phosphorylating particular DNA replication machinery components, such as CDC6 [4, 5]. Approaching late S phase, Cyclin A-CDK1 kinase activity is augmented, which coordinates with Cyclin A-CDK2 in G2 phase to promote mitotic entry [6–8]. The abundance of Cyclin B accumulates in M phase, resulting in Cyclin B-CDK1 complex activation and mitosis progression [9–11]. In addition to the fluctuating accumulation of cyclin activators, two families of CKIs, namely INK4 and CIP/KIP, also contribute to the periodic activation of CDKs over the course of the cell cycle. Briefly, the INK4 proteins (inhibitors of CDK4) specifically inhibit the catalytic subunit of CDK4 and CDK6, dephosphorylating RB1 and rendering its inhibitory effect on E2F transcription factors, while inhibitors of the CIP/KIP family have relatively more broad effects by modulating the kinase activities of Cyclin A-, B- and E-dependent kinases [12]. It is well characterized that the removal of cyclins is tightly regulated by the ubiquitin pathway and thus governs cell cycle progression in a time-efficient manner [13]. Moreover, the negative regulators of cyclin-CDK complex (CKIs), such as p21 and p27, have also been shown to be targeted for proteasomal degradation [14–16]. Altogether, these findings demonstrate that the cell cycle progression is predominantly regulated by the ubiquitin–proteasome system [17, 18].

**Fig. 2:**
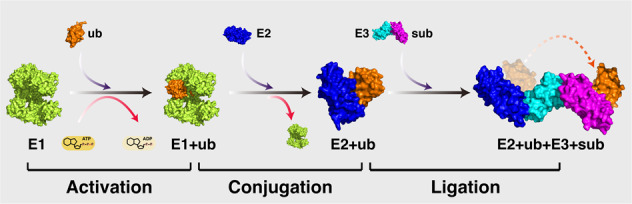
A schematic diagram of the ubiquitination process. Ubiquitination is an enzyme-mediated posttranslational modification by which the ubiquitin protein is attached to a substrate protein. This process involves three main steps: (1) activation step, the ubiquitin protein is activated by an E1 ubiquitin-activating enzyme, with ATP providing energy; (2) conjugation step, the ubiquitin protein is transferred from E1 to the active site of an E2 ubiquitin-conjugating enzyme; (3) ligation step, the ubiquitin protein is attached to the substrate (sub) with the catalyzation of an E3 ubiquitin ligase. Protein structures used here are as follows: Ub (1UBQ); E1–Ub complex (6DC6); E2–E3–Ub complex (4AP4).

**Fig. 3:**
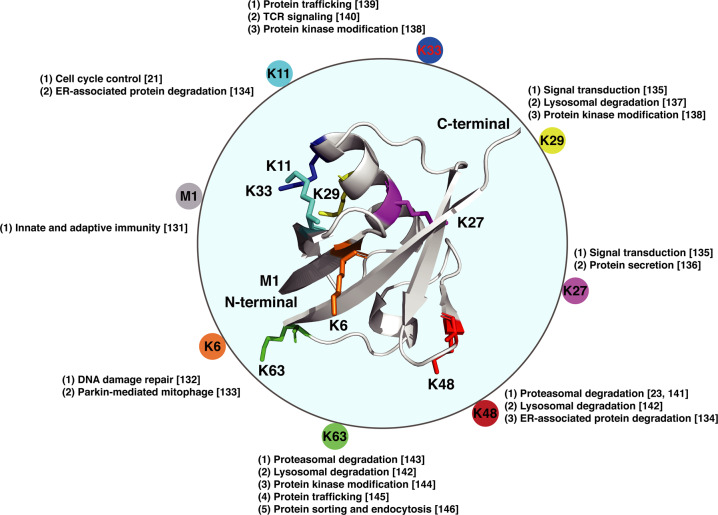
Molecular structure of the ubiquitin molecule and linkage-dependent function of ubiquitination. Ubiquitin is a small protein (8.6 kDa) that is expressed in all eukaryotic cells. There are eight amino acids (the N-terminal methionine M1 and seven lysine residues: K6, K11, K27, K29, K33, K48, and K63) that can serve as docking points for additional ubiquitin addition. The ubiquitination can be either a single ubiquitin protein (monoubiquitination) or a chain of ubiquitin (polyubiquitination). The variety of different modifications confers the diversity of linkage-dependent function of ubiquitination. Structure of ubiquitin is 1UBQ.

Ubiquitin is an ubiquitously expressed small regulatory protein in living cells [19]. The addition of ubiquitin to a substrate protein is called ubiquitination, which is catalyzed by three types of enzymes, ubiquitin-activating enzymes (E1s), ubiquitin-conjugating enzymes (E2s), and ubiquitin ligases (E3s), involving three major steps [20]. Briefly, the ubiquitin protein is first activated by E1-mediated catalysis of the acyl-adenylation of the C-terminus of the ubiquitin protein, followed by transferring ubiquitin to an active cysteine residue in the context of ATP providing energy. Then, E2 ubiquitin-conjugating enzymes catalyze the transfer of the ubiquitin from E1 to the active site cysteine of E2. Finally, an E3 ubiquitin ligase brings the substrate and ubiquitin-loaded E2 together, catalyzing the transfer of the ubiquitin from E2 to the substrate (Fig. 2). The ubiquitination process can involve either a single ubiquitin protein (monoubiquitination) or a chain of ubiquitin linked via different lysine residues of the ubiquitin molecule (termed as polyubiquitination). As ubiquitin possesses seven lysine residues (K6, K11, K27, K29, K33, K48, and K63) and one N-terminal methionine (M1) that can serve as docking points of additional ubiquitin chain formation, polyubiquitination of target protein exhibits distinct functional consequences depending on the lysine residue of the ubiquitin that is linked (Fig. 3).

Briefly, K11-linked ubiquitin chain was found to regulate the substrates of the anaphase-promoting complex/cyclosome (APC/C) complex and control progression through mitosis, while Skp1-Cul1-F-box (SCF) ubiquitin ligase complex catalyzed K48-linked polyubiquitination and subsequent proteasomal degradation of substrates to modulate cell cycle progression [21–23]. To gain more insights into the functional diversity and specificity of linear-, mono- and linkage-dependent polyubiquitination modification, readers are encouraged to refer to the extensive literature which has been summarized previously [24–26]. Here, we will mainly focus on summarizing the physiological role of the ubiquitin signaling in cell cycle control and tumorigenesis, with primary purpose to provide a better understanding of ubiquitination-mediated cell cycle regulation and ubiquitin ligase targeted anticancer therapies.

## Overview of the function of APC/C and SCF E3 ligases in modulating cell cycle progression

Progression through the cell cycle is determined by phosphorylation of CDK substrates [27, 28]. To ensure the cell cycle progression occurs in an ordered manner, the oscillating activity of CDKs is established and tightly orchestrated by multiple mechanisms including transcription, phosphorylation, as well as periodic degradation of their cyclin coactivators and CKIs as mentioned above [13–16, 29]. Of note, the proteolytic degradation of regulators of CDKs is primarily controlled by two families of E3 ubiquitin ligases in mammalian cells, APC/C, and SCF protein complex [30].

**Fig. 4:**
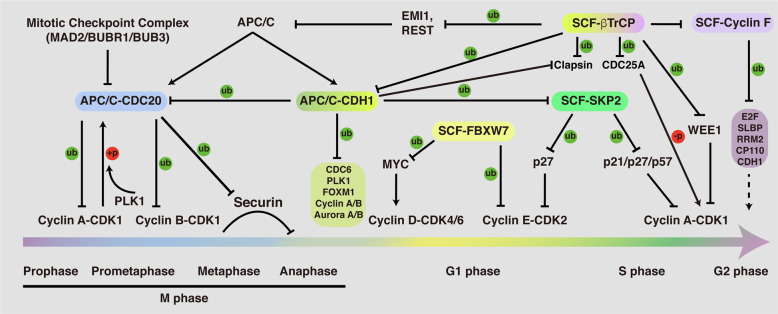
Function and regulation of the APC/C and SCF E3 ligases throughout the cell cycle. APC/C and SCF E3 ligases serve as the two important types of E3 enzymes to regulate the cell cycle progression. Briefly, APC/C-CDC20 functions in prophase to metaphase to mediate the ubiquitination and proteasomal destruction of Cyclin A/B, Securin; APC/C-CDH1 functions from anaphase to G1 phase to modulate the protein stability of CDC6, PLK1, FOXM1, Cyclin A/B, and Aurora A/B, ensuring M phase progression and G1 phase maintenance. Four F-box proteins of SCF E3 ligase complex have been well-documented to function in regulation of the cell cycle progression: FBXW7, βTrCP, SKP2, and Cyclin F. FBXW7 functions largely as a tumor suppressor to mediate the protein destruction of MYC and Cyclin E. By contrast, SKP2 is believed to serve as an oncogene, which mediates the ubiquitination and degradation of CDK inhibitors, such as p21, p27, and p57. βTrCP is found to play a dual role in controlling CDK1 activity, turning it on by inducing Claspin and WEE1 degradation in G2 phase, and turning it off by inducing the degradation of CDC25A, EMI1, and REST. Cyclin F functions in G2 phase to restrict the activity of E2F, the synthesis of replicative histones (SLBP), and the levels of ribonucleotides (RRM2), as well as regulate centrosomal duplication (CP110). In addition, the APC/C and SCF E3 ligases control each other. For example, CDH1 mediates the degradation of SKP2 in G1 phase, while βTrCP and Cyclin F were reported to control the destruction of CDH1. Moreover, CDH1 is responsible for mediating CDC20 for proteasomal degradation, while the protein stability of Cyclin F was shown to be controlled by βTrCP.

The APC/C is a multi-subunit cullin-RING E3 ubiquitin ligase that functions in mitotic phase and G1 phase, regulating cell cycle progression through M phase and entry into S phase [31, 32]. The temporal regulation of APC/C activity is prominently achieved through combination of two structurally relevant coactivators, CDC20 and CDH1, which are sequentially activated to regulate mitotic progress and G1 stabilization. Briefly, mitotic phosphorylation of APC1 relieves its auto-inhibition and promotes APC/C activation by facilitating CDC20 engagement [33, 34]. Activation of APC/C^CDC20^ then mediates the proteasomal degradation of Cyclin B1 and Securin, facilitating chromosome segregation and anaphase onset [31, 35, 36]. In addition, degradation of Cyclin B1 inactivates CDK1, preventing APC/C-CDC20 combination while releasing its inhibitory phosphorylation of CDH1 [37]. Simultaneously, CDC14 is released and activated with the onset of anaphase, dephosphorylating and activating the APC/C^CDH1^ E3 ligase [38, 39]. Together, suppression of CDK1 and activation of CDC14 build up a swift transition from APC/C^CDC20^ to APC/C^CDH1^ during anaphase. Activation of CDH1 then mediates a large number of mitotic and G1 regulators for ubiquitination and proteasomal degradation, such as Cyclin B1, PLK1, CDC20, FOXM1, and SKP2, facilitating irreversible mitotic exit and G1 maintenance [40]. As cells reach late G1 phase, multiple mechanisms are then employed, such as CDK-mediated phosphorylation, degradation of the E2 enzyme UBE2C, and accumulation of pseudo-substrate EMI1, to inactivate CDH1 to facilitate G1/S transition [40]. Collectively, our current knowledge suggests that the APC/C is mainly active from mitosis through late G1 phase over the course of the cycle (Fig. 4).

The SCF complex contains three core subunits Cullin, SKP1, and RBX1, as well as a variable F-box protein. In comparison to APC/C, the number of substrates of SCF complex is enormous due to the variety of F-box proteins. Although almost 70 F-box proteins have been reported in mammals [41], only four of them, SKP2, FBXW7, βTrCP, and Cyclin F, have been well characterized in the cell cycle regulation [42–44].

In the early G1 phase, the E3 ligase activity of SKP2 is suppressed due to the active presence of APC/C^CDH1^ [45, 46]. However, when cells approach late G1 phase, the enzymatic activity of CDH1 is diminished and phosphorylation of SKP2 by Cyclin E-CDK2 protects it from APC/C^CDH1^-mediated proteasomal degradation, conferring activation of the SCF^SKP2^ E3 ligase complex [40, 47]. Meanwhile, the activated Cyclin E-CDK2 complex mediates phosphorylation and ubiquitination of p27 [48, 49]. Subsequently, the phosphorylated p27 is recognized and ubiquitinated by SKP2, leading to its proteasomal degradation [50]. Consequently, degradation of p27 relieves its suppression on Cyclin E-CDK2, leading to a positive feedback loop which contributes to RB1 full phosphorylation and the G1/S transition [2, 3]. In addition to p27, the proteolytic degradation of the other two CIP/KIP members, p21 and p57, is also controlled by SKP2 [51, 52]. Given the importance of the CIP/KIP family of CKIs in regulating cell cycle transition [12, 53], it is conceivable that the disruption of SKP2 E3 ligase activity would cause dysregulation of cell cycle progression. As mentioned above, Cyclin A association with CDK2, in replace of Cyclin E, is involved in the regulation of the initiation of DNA synthesis when cells enter S phase [4, 5]. Thus, the timely removal of free Cyclin E is necessary to ensure cell progresses forward through the cell cycle. In support of this notion, SKP2 was found to be capable of ubiquitinating free Cyclin E for proteasomal degradation [54].

When cells entry into G2 phase, Cyclin F ubiquitinates and restricts the activity of E2Fs, the main and most critical transcriptional engines of the cell cycle [55, 56]; mediates degradation of SLBP to limit H2A.X accumulation and apoptosis upon genotoxic stress [57]; controls genome integrity and centrosome homeostasis by degrading Ribonucleotide Reductase M2 (RRM2) and CP110, respectively [58, 59]. Interestingly, the protein stability of Cyclin F is modulated by βTrCP to control timely mitotic progression [60].

During the early stage of mitosis, Cyclin A associates and activates CDK1, driving the initiation of chromosome condensation [61–63]. Once the activity of APC/C^CDC20^ is turned on in prometaphase, Cyclin A is ubiquitinated and degraded by the proteasome [64, 65]. Of note, destruction of Cyclin B, another crucial mitotic cyclin that can be targeted by the APC/C^CDC20^ for proteasomal degradation, is initiated during the metaphase, and occurs significantly later than the destruction of Cyclin A [66, 67]. Investigation of this difference of temporal degradation between Cyclin A and Cyclin B suggests that destruction of Cyclin A is likely spindle checkpoint independent, while the proteolytic degradation of Cyclin B1 is largely sensitive to the spindle assembly checkpoint (SAC) [68, 69]. Therefore, degradation of Cyclin B1 by APC/C^CDC20^ is blocked by MAD2 in prometaphase when chromosomes are not fully attached to the mitotic spindles [70, 71]. Moreover, protein stability of CDT2 was found to be regulated by FBXO11 ubiquitin ligase [72]. Collectively, the tightly orchestrated sequential destruction of mitotic regulators contributes to dictate the timing of events during mitotic exit.

**Fig. 5:**
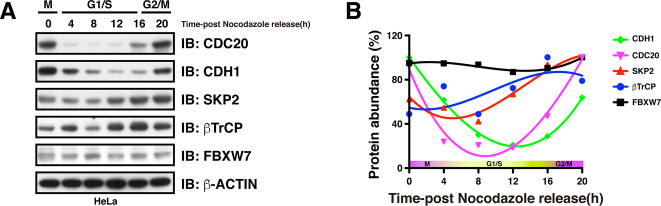
Protein accumulation profiles of APC/C and SCF adapter proteins during the cell cycle. **A** Western blot showing the protein abundance of APC/C and SCF adapter proteins over the course of the cell cycle. **B** Quantification of protein density showing the accumulation of protein levels of APC/C and SCF adapter proteins throughout the cell cycle.

 The protein abundance of CDH1 diminished during late G1 phase (Fig. 5), which is partially mediated by βTrCP- and Cyclin F-directed proteasomal degradation [73, 74]. Moreover, βTrCP has been found to play dual roles in controlling CDK1 activity, turning it on by inducing WEE1 and Claspin degradation during G2 phase [75, 76], and turning it off by inducing the degradation of EMI1 and CDC25A in M and S phase, respectively [77, 78]. Activation of CDK1 in G2 phase phosphorylates EMI1, priming it for recognition and degradation by βTrCP, ensuring the timely activation of APC/C [77, 79]. In addition, βTrCP also controls APC/C E3 ligase activity in part by mediating the degradation of REST, a repressor of MAD2 transcription [80]. In addition to the well-established SKP2, βTrCP, FBXW7, and Cyclin F, roles of other F-box proteins involved in regulating cell cycle progression have been summarized elsewhere [81].

## Role of ubiquitination signaling in cell cycle checkpoints

It is well established that three checkpoints operate in eukaryotic cells to ensure ordered and accurate cell cycle progression (Fig. 1). In addition to the roles of ubiquitination in cell cycle regulation mentioned above, ubiquitin signaling is also involved in mediating cell cycle checkpoint response. In the G1/S checkpoint, phosphorylation and degradation of CDH1 are required to release its inhibition on SKP2, allowing p27 destruction and consequent Cyclin E-CDK2 activation. The G2/M checkpoint prevents cells initiating mitosis in the context of damaged or incompletely replicated DNA. Upon DNA damage, activation of ATR phosphorylates and activates CHK1 protein kinase, which then mediates phosphorylation and proteasomal degradation of CDC25A in a SCF^βTrCP^-dependent manner [82, 83]. Suppression of CDC25A prevents CDK1 from dephosphorylation and activation, arresting cells in G2 phase for sufficient DNA damage repair [83, 84]. Moreover, CHK1-mediated phosphorylation of RAD51 counteracts EMI1-dependent degradation, thereby restoring RAD51-dependent homologous recombination (HR) repair [85]. Of note, recent findings showed that DNA damage-induced activation of ATM phosphorylates p53 and facilitates its binding with FBXW7, leading to subsequent p53 ubiquitination and proteasomal degradation [86]. In addition to the ubiquitination of key cell cycle regulators, histone ubiquitination also plays crucial roles in DNA damage response and cell cycle advance. For example, site-specific ubiquitination of H2A organizes the spatio-temporal recruitment of DNA repair factors to contribute to DNA repair pathway choice between homologous recombination (HR) and non-homologous end joining (NHEJ) [87], while deubiquitination of H2A is required for chromosome segregation when cells enter mitosis [88]. The M checkpoint is also known as spindle assembly checkpoint (SAC), by which cells assess whether all chromosomes are properly attached to the spindle. In the context of chromatids being misplaced, kinetochores activate the SAC, which then inhibits the E3 ligase activity of APC^CDC20^ and delays cell division until accurate chromosome segregation can be guaranteed [89]. By contrast, once all chromosomes are correctly attached to the microtubule spindle apparatus, APC^CDC20^ mediates Cyclin B1 and Securin for ubiquitination and proteasomal degradation, allowing for chromosome segregation and metaphase-to-anaphase transition [31, 35, 36].

## Substrate recognition by APC/C and SCF E3 ligase

Recognition of the substrates by corresponding E3 ligases is achieved by short destruction-mediating sequence elements, which is named degron [90]. The best-studied degron in targets of APC/C are the nine-amino acid destruction box (D-box: RxxLxxxxN) and the KEN box (KENxxxN), which are preferred by CDH1 and CDC20 or CDH1, respectively [13, 91] (Table 1). Nonetheless, a spectrum of other amino acid sequences has also been found to be recognized by the APC/C complex, such as the ABBA motif ([ILVF]x[ILMVP][FHY]x[DE]) which was identified in Cyclin A, BUBR1, BUB1, and Acm1 [92]. In comparison to APC/C, F-box proteins recognize their substrates in multiple ways, among which the best-characterized F-box proteins bind to phosphodegrons in their substrates [93]. Thus, phosphorylation of the substrates plays an important role for F-box protein-mediated recognition and ubiquitination. βTrCP recognizes the DSGxxS/T degron  in which the serine residues or serine and threonine residues are phosphorylated  [93, 94] (Table 1). For example, CDK1 phosphorylation of the DSG degron of EMI1 primes its recognition and destruction by βTrCP to activate APC/C complex [77, 95]. Substrates of FBXW7 usually contain a canonical degron S/TPPxS/T [93, 96] (Table 1). Serving as an example, CDK2 phosphorylation of the TPPxS of Cyclin E determines its recognition and ubiquitination by FBXW7 [97, 98]. Unlike βTrCP and FBXW7, SKP2-dependent ubiquitination and degradation of CKIs, such as p27, requires not only the CDK-mediated phosphorylation, but also an accessory protein, CKS1, representing a cofactor-dependent substrate recognition [48–50, 99]. Cyclin F contains three separate modules, the pseudo-catalytic, substrate recruitment, and regulatory modules. It was reported to utilize the hydrophobic patch in the cyclin domain to bind the CY-containing substrates [44]. Mechanisms and functions of substrate recognition by F-box protein have been extensively summarized in [44, 93].

**Table 1:** Examples of key APC/C and SCF substrates involved in cell cycle control.

E3	Adapter	Substrate	Degron	Gene function	Role in cancer	Refs.
APC/C	CDC20	Cyclin A	D-box (RxxLxxxxN)	CDK1/2 activation and G1/S, G2/M transition	Oncogenic	[69]
		Cyclin B1		CDK1 activation and mitosis progression	Oncogenic	[66, 67]
		Securin		Inhibition of chromosome segregation and p53 activity	Oncogene	[31]
	CDH1	Aurora A	D-box (RxxLxxxxN) or KEN-box (KENxxxN)	Regulation of mitosis progression	Oncogene	[147]
		CDC20		Activator of APC/C complex	Oncogenic	[91]
		PLK1		Regulation of mitosis progression	Oncogene	[148]
		SKP2		Substrate recognition component of SCF E3 ligase	Oncogene	[45, 46]
		FOXM1		Transcription factor involved in DNA replication and mitosis	Oncogene	[149]
SCF	SKP2	p21/p27/p57	N/A	CDK inhibitor	Tumor suppressor	[50–52]
		p130		Transcription factor regulating cell cycle entry	Tumor suppressor	[111]
		CDT1		Regulator of DNA replication and mitosis	Oncogenic	[112]
	FBXW7	MYC	S/TPPxS/T	Transcription factor	Oncogene	[115]
		Cyclin E		CDK2 activation and G1/S transition	Oncogenic	[97, 98]
		JUN		Transcription factor	Oncogene	[150]
	βTrCP	FOXO3	DSGxxS/T	Transcription factor	Tumor suppressor	[119]
		WEE1		CDK1 inhibition	Oncogenic	[75]
		CDC25A		CDK1 activation	Oncogenic	[78, 82]
		EMI1		Regulator of APC activity	Oncogenic	[77, 79]
	Cyclin F	SLBP	CY motif	Histone pre-mRNA processing	Not defined	[57]
		RRM2		Catalyzes the biosynthesis of deoxyribonucleotides	Oncogene	[58]
		CP110		Necessary for centrosome duplication	Not defined	[59]

## Role of APC/C and SCF E3 ligases in tumorigenesis

Accumulating evidence have shown that CDC20 is frequently overexpressed in a wide range of cancers, indicating that it might function as an oncoprotein [32, 100]. From the perspective of cell cycle, degradation of Cyclin B and Securin is required for the onset of anaphase [35, 101]. It is thus conceivable that the loss of CDC20 causes metaphase arrest in mouse embryos [102]. In support of the oncogenic role, genetic ablation of *CDC20* results in efficient tumor regression [103], while the loss of CDC20 inhibition promotes tumorigenesis [104], advocating CDC20 as a potential therapeutic target for cancer treatment [105]. In contrast, CDH1 has been found to be downregulated in a large variety of human cancers [32, 100]. *CDH1*-deficient cells proliferate inefficiently and *CDH1* heterozygous animals show increased susceptibility to spontaneous tumors, largely conferring CDH1 a tumor suppressor role [106]. In addition, accumulation of SKP2 due to the loss of *CDH1* is considered to promote proteasomal degradation of CIP/KIP family of CKIs and thus facilitate tumorigenesis.

Regarding the role of SCF E3 complex in cancer development, emerging evidence suggest that it acts in a F-box protein- and context-dependent manner [107–109]. Specifically, SKP2 is a well-defined oncoprotein and was found to be overexpressed in various human cancers [109, 110]. Targets of SKP2 are mainly tumor suppressor proteins including p21, p27, p57, p130, and CDT1 [50–52, 111, 112]. Therefore, SKP2 exerts its oncogenic function mainly through degradation of its tumor suppressive targets. In support of the oncogenic role of SKP2, pharmacological inhibition of SKP2 was found to be able to restrict cancer progression [113]. In contrast to SKP2, FBXW7 is believed to function mainly as a tumor suppressor by targeting various oncogenic proteins for degradation [96, 107–109]. For example, proteasomal destruction of Cyclin E through FBXW7-mediated ubiquitination blocks CDK2 activation in late G1 phase and thus delays G1/S transition, arresting cells in G1 phase [97, 98, 114]. Another well-established oncogenic substrate of FBXW7 is MYC [115], which serves as a transcription factor involved in the genesis of many human cancers [116]. Regarding Cyclin F, it is believed to function as a tumor suppressor by controlling genome integrity and centrosome duplication by regulating the protein stability of RRM2 and CP110, respectively [44, 58, 59]. Looking at the substrate list of βTrCP, it is obvious that βTrCP plays a dual role in regulating CDK1 activity, turning it on by inducing WEE1 and Claspin destruction [75, 76], while turning it off by targeting EMI1 and CDC25A for proteasomal degradation [77, 78]. Importantly, preclinical studies have validated WEE1 inhibition as a viable therapeutic target in treating cancer [117], and CDC25A is also deemed as a suitable therapeutic target for cancer treatment [118], establishing βTrCP as a tumor suppressor. On the other hand, βTrCP was found to be involved in mediating the proteasomal degradation of tumor suppressors, such as FOXO3 and DEPTOR [119, 120]. Taking these results into consideration, βTrCP might be expected to be oncogenic and exert a tumor suppressive role in a context-dependent manner [107, 109].

## Conclusion and perspective

In the present review, we mainly summarized the proteolytic signals involved in the cell cycle control. Moreover, non-proteolytic ubiquitination of the cell cycle regulators also plays crucial roles in controlling cell cycle progression. For example, the endoplasmic reticulum lipid associated protein 2 was found to interact and facilitate K63-linked ubiquitination and stabilization of Cyclin B1, facilitating mitosis exit [121]. Di-ubiquitination of the minichromosome maintenance protein 10 is required for its interaction with PCNA to facilitate DNA elongation in S phase [122]. Although accumulating evidence supporting critical roles of non-proteolytic ubiquitin signals in regulating cell cycle progression, unlike the functions of proteolytic ubiquitin signals which have been studied extensively, relatively little is known regarding the roles and mechanisms underlying cell cycle control that go beyond proteasomal degradation. In addition, it is well characterized that ubiquitination is a reversible process as the ubiquitin can be removed from the modified proteins by an array of deubiquitinating enzymes (DUBs) [123]. Of note, DUBs have been found to play critical roles in regulation of mitosis [124], and small molecular inhibitors against DUBs are expected to offer novel therapeutic opportunities for cancer treatment [125]. However, the roles and substrates of DUBs in regulating cell cycle events remain not well understood. In particular, how is the balance of ubiquitination–deubiquitination achieved to ensure accurate cell cycle progression remains elusive and needs additional in-depth investigations.

With respect to the well-established APC/C E3 ligase complex, activation of CDC20 is required for anaphase onset, while CDH1 plays a central role in mediating mitosis exit and G1 maintenance. The ordered activation of CDC20 and CDH1 is essential for accurate mitosis progression. Mitotic phosphorylation of APC/C relieves its auto-inhibition and facilitates CDC20 engagement [33, 34], while BUB1-mediated phosphorylation of CDC20 upon spindle checkpoint activation inhibits the ubiquitin ligase activity of APC^CDC20^, ensuring the fidelity of chromosome segregation [126]. Although the protein stability of CDH1 has been reported to be modulated by βTrCP and Cyclin F [73, 74], regulation of CDH1 E3 ligase activity is not well known yet. A previous study has shown that there are 19 serine and threonine residues on CDH1 that can be phosphorylated by multi-kinases in vivo, indicating that the phosphoregulation of CDH1 is much more complex [127]. Another intriguing phenomenon is about the timing of degradation of proteins controlled by the same substrate adapters. We know that CDC20 functions as an upstream adapter protein for Cyclin A, Cyclin B, and Securin, mediating their ubiquitination and proteasomal degradation during mitosis. Interestingly, degradation of Cyclin A proceeds before that of Cyclin B and Securin, which is governed by the presence of spindle checkpoint signaling [128]. However, the detailed mechanisms underlying the spindle checkpoint imposed various E3 ligase activities of CDC20 toward different substrates need to be further investigated.

Overall, the cell cycle progression is tightly regulated to ensure the genomic integrity and identity in daughter cells, and ubiquitin signaling involves almost each step of the cell cycle. Dysregulation of the ubiquitination modification led to uncontrolled cell cycle progression and eventually resulted in tumorigenesis [18]. Based upon this notion, targeting the ubiquitin system has provided effective therapeutic strategies for cancer treatment [129–146]. At the moment, how these signaling events are integrated and orchestrated in a time–space-dependent manner remains not fully understood. In addition, only a handful of drugs targeting the ubiquitin system have been approved by the FDA. Therefore, a better understanding of the ubiquitin signaling in cell cycle control will expand and diversify the range of anticancer strategies and benefit the clinical treatment of cancer patients in the future.
